# Attitudinal and perceptual factors in body image distortion: an exploratory study in patients with anorexia nervosa

**DOI:** 10.1186/2050-2974-1-17

**Published:** 2013-05-03

**Authors:** Amanda Waldman, Rachel Loomes, Victoria A Mountford, Kate Tchanturia

**Affiliations:** 1 King’s College London, Division of Psychological Medicine, Institute of Psychiatry, London, UK; 2 South London and Maudsley NHS Foundation Trust, London, UK

**Keywords:** Anorexia nervosa, Eating disorder, Body image, Haptic perception, Perfectionism, Body dissatisfaction

## Abstract

**Background:**

Body image disturbance is a core feature of anorexia nervosa (AN). Attitudinal and cognitive biases as well as fundamental perceptual differences have been hypothesized to play a role in this disturbance.

**Method:**

This study investigated body image dissatisfaction and distortion, haptic perception and perfectionism in 30 patients with AN and 31 age-matched healthy controls. Participants completed perceptual tasks and self-report measures.

**Results:**

As predicted, participants with AN scored significantly higher on body dissatisfaction, perfectionism measures and had greater body distortion (as assessed by a body size estimation task). Cognitive–affective factors and perfectionism were highly correlated with body image distortion in AN. No significant differences were found between groups on the generic perception task.

**Conclusions:**

Findings did not confirm the hypothesis of fundamental perceptual inefficiencies in body image disturbance in individuals with AN. Despite renewed interest in fundamental perceptual factors implicated in body image disturbance, these findings suggest that it continues to be important to focus treatment on cognitive affective biases versus fundamental perceptual inefficiencies.

## Background

Body image disturbance is one of the diagnostic criteria [1] for Anorexia Nervosa (AN), and has been implicated in both the development [2,3] and maintenance [4] of eating disorders. Furthermore, body image disturbances often persist following recovery (e.g. [5]) and predict relapse [6]. Such disturbance can manifest as disturbance of percept (i.e. distortion) and concept (i.e. dissatisfaction).

A growing consensus exists that two types of body representation may be impaired in AN: the body schema and the body image (for reviews: [7,8]). The body schema is defined as a sensorimotor representation of the body in action – whether this action is actual, anticipated or imagined [9]. The body image, by contrast, consists of several components, subdivided into the perceptual (sensory perception) and the attitudinal (cognitive and affective factors). These body image representations are not used for action, though may influence and be influenced by the body schema.

Distortions in either the body schema or the body image could result in body size overestimation. Recent research in eating disorders has focused on attitudinal components of body image disturbance, with individuals with AN showing higher levels of body dissatisfaction than healthy controls ([10]; see [11] for a review). However, there has been a resurgence of interest in the role of perceptual disturbances of body representations. Benninghoven et al. [12] found increased body size overestimation in women with eating disorders, but no impairment of estimation of other women’s bodies, nor of male ideals of female body attractiveness, concluding that body distortion was confined to the processing of ‘self-referential’ information, rather than body image information in general. Further, there have been findings of sub-optimal visuo-spatial performance in individuals with AN, on subtests of the Wechsler Adult Intelligence Scale [13] and the Rey–Osterreith Complex Figure (ROCF; Meyers &[14]). In some, these difficulties persist after weight recovery (e.g. [15]). Visuo-spatial performance has also been shown to correlate with body size estimation in the general population using the Adjustable Light Beam Apparatus [16]. However, visual body image disturbance has also been found to result from distortions of memory rather than perception [17], supporting the role of attitudinal rather than perceptual influences on body image disturbance. Interoceptive awareness (bodily perception of physiological sensations) may also be impaired in AN (e.g. [18]).

Another form of bodily awareness, haptic perception (a form of tactile perception), is defined as the recognition of external stimuli, through the combination of somatosensory and proprioceptive perception [19]. Haptic perceptual impairment has been noted in individuals with AN, with inefficiencies in haptic set shifting tasks [20,21]. Childhood perfectionism is also associated with sub-optimal performance on neuropsychological measures of set-shifting (i.e. difficulties in responding effectively to rule changes) [20,22,23] and is a personality trait linked to an increased risk of developing an eating disorder [24].

Further haptic-perceptual impairment was noticed in somatosensory tasks [25] persisting across states of illness and recovery, suggesting that this may be a trait factor in the disorder. Grunwald et al. [26] also found impairment in performance on a bimanual somatosensory task, the Angle Paradigm Task, in adolescents with AN. The impaired performance was only significant on right-handed tasks, which Grunwald and colleagues related to a right parietal lobe dysfunction, using the direct access theory of perceptual processing [27]. Differential activation of the parietal cortex has been implicated in the presentation of AN (e.g. [28]) and is the area of the brain most consistently associated with the disorder in the functional neuroimaging literature [29].

The parietal lobe is also thought to be responsible for the integration of proprioceptive and visual information regarding one’s own body, with this integration forming the basis of the physical body representation (schema; [30]). Disorders of body image have been associated with both left and right parietal lobes, but little is known about the neural correlates of body image in AN, particularly with regard to laterality. Some have found differential activation in the left hemisphere [17,31], and others the right hemisphere (e.g. [32]). Neuroimaging studies investigating body dissatisfaction and distortion in AN have found links with parietal lobe function [31,33]. Indirect associations with parietal lobe dysfunction have been postulated in studies which found sub-optimal visual and tactile performance in AN [10,34], but no study, to our knowledge, has yet explored the interactions of haptic perception, body image dissatisfaction and distortion together in relation to perfectionism.

This study therefore first explored haptic perceptual task performance in individuals with a current diagnosis of AN as compared to age-matched controls; secondly, the attitudinal and perceptual aspects of body image disturbance, and their relative contribution to body image distortion in AN were investigated.

The following hypotheses were tested: 1) individuals with AN would make greater body size estimation (BSE) errors than healthy controls (HC); 2) individuals with AN would have greater body dissatisfaction than HC; 3) individuals with AN would perform worse than HC on a measure of haptic perception; 4) the AN group would score more highly on measures of perfectionism compared to HC individuals; and 5) correlations would exist between attitudes to body image, haptic perception, perfectionism and distorted body image in the AN group.

## Method

### Participants

Sixty-one adult females were recruited to the study. Thirty females with a clinical diagnosis of AN or EDNOS-AN were recruited from eating disorder services of hospitals in South London. Diagnosis was made by trained clinicians using a semi-structured interview schedule and DSM-IV criteria. Thirty-one age-matched females were recruited to the HC group. A screening questionnaire confirmed the absence of diagnosed eating disorders and other psychiatric disorders. Study volunteers with an intellectual disability, head injury or non-fluent English were excluded from the study.

### Materials and procedure

#### Procedure

Participants were asked to read the information sheet, sign the consent form and complete the screening form. The study was approved by a National Health Service ethics committee. The self-report measures were completed followed by the perceptual tasks.

### Self-Report/attitudinal measures

*The Silhouettes Body Perception Scale*[35] is an attitudinal measure of body image, using a scale of silhouettes increasing in size. Participants mark the silhouette that best corresponds with their current body shape, in their view, and the silhouette that matches how they would wish to look. To measure the level of body dissatisfaction, the score from item 2 ‘how you would wish to look’ is subtracted from the score from item 1 ‘your current body shape’. A score of 0 represents no body dissatisfaction, a positive number score represents a desire for a smaller body shape and a negative number score represents a desire for a larger body shape.

The scale has satisfactory test-retest reliability (*r* = .82) and convergent validity (*r* = .73) in multi-ethnic general populations, with acceptable discriminative power in differentiating between samples [35].

*The Body Image Avoidance Questionnaire* (BIAQ; [36]) is a nineteen-item self-report behavioural measure of body image, assessing behaviours associated with a negative body image, such as avoidance of situations that provoke concern about physical appearance. Participants respond on a scale of 0 (never) to 5 (always). In this study it was used as an additional measure of body dissatisfaction.

The BIAQ has proven psychometric properties in clinical and non-clinical populations [36] with good internal consistency (Cronbach’s alpha of .89), as well as showing modest associations with body size estimation tasks (*r* = .22, *p* < .01), strong associations with negative attitudes to weight and shape (*r* = .78, *p* < .0001). Furthermore, it distinguishes satisfactorily between clinical and non-clinical populations [36], as well as responding to change resulting from treatment of eating disorders.

*The Eating Disorders Examination-Questionnaire* (EDE-Q; [37]) is a self-report measure of eating disorder symptomatology. It consists of twenty-eight items, asking participants to mark to which degree they have engaged in each behaviour over the previous 28 days. The questionnaire predominantly consists of scale items of 0-6, with a higher score indicating a greater intensity or frequency. There are four subscales: Dietary Restraint, Eating Concern, Weight Concern, and Shape Concern. The Weight and Shape Concern subscales were used in this study as a measure of body image.

The EDE-Q’s internal consistency (Cronbach’s α = .78 - .93), temporal stability and test-retest reliability (*r* = .81- .94) have been established in assessing the core attitudinal features of eating disorders [38-40]. It has been validated as a screening tool to detect eating disorders in community samples [39] and in primary care [41].

*The Frost Multi-dimensional Perfectionism Scale* (FMPS; [42]) is a self-report, 35-item, multi-dimensional measure of perfectionist traits, which generates an overall perfectionism score. Scores for six subscales reflecting various domains of perfectionism can also be calculated: concern over mistakes, doubts about actions, personal standards, parental expectations, parental criticism and organization. The total perfectionism score is the sum of all subscales. Frost et al. [42,43] have reported good reliability of the subscales (Cronbach’s α = .77 to .93) and good concurrent validity with other perfectionism scales as well as good construct validity in relation to a variety of measures of psychopathology. Strong validity was also shown by Enns & Cox [44]. This measure is also widely reported in the eating disorder literature (e.g. [45]).

### Perceptual measures

*The Adjustable Light Beam Apparatus* (ALBA; [46]) is an experimental task measuring an individual’s accuracy in estimating ones own body size. An overhead projector, with the apparatus attached, is placed at 1.5 metres from a blank wall. Participants adjust rods on the apparatus to beam rays of light on to the wall. Each beam of light is used to approximate the width of four body parts in turn (cheeks, waist, hips and thighs). A type of silhouette is created, the entirety of which can be adjusted if desired. Measurements of the width of the beam were taken from the wall, and secondary measurements of the width of the gap on the apparatus itself were also taken. This second measurement is converted to a full-scale measurement using the formula outlined in the original paper [46]. Actual measurements of the waist, hips and thighs were then taken using calipers. Cheek measurements were not taken, as this anchor point is used as a practice item, and was not found to correlate well with the other body sites, in the original study. The two sets of recordings were compared to provide a calculation of accuracy in BSE. The measure has shown good test-retest reliability in women, in terms of constancy of overestimation over time (over 1 week) and discriminant validity between size estimation and size dissatisfaction, as well as between size estimation and overall satisfaction with appearance.

*The Angle Paradigm Task*[26] is a sensorimotor perception task, measuring haptic perception. In this bimanual task, one metal rod is always placed at 90° and the other rod placed at another angle. Participants were blindfolded and adjusted the first metal bar, set at 90°, so that it was either parallel or mirror image to the second bar. There were four conditions in the task: adjusting the angle as a parallel with the right hand, and then with the left hand, and adjusting the angle as a mirror image with the right hand, and then with the left hand. For each condition there were five trials, with the metal bar set at 45°, 22°, 65°, 15° and 35°. Before each condition participants were given the opportunity to practice the task once with the blindfold on, but without any visual feedback. The outcome measure for the task was the difference between the angles of the set and adjusted bars. The mean of the total time taken to adjust each angle was used as an additional measure of perfectionism, as in the original paper. Left-handed individuals are excluded from this task, as the original study looked at only right-handed individuals. The task is intended to explore the theorised right parietal dysfunction in AN, and it is posited that a left-handed individual is not likely to experience the same demands on the right parietal cortex in this task as a right-handed individual.

The original authors were able to detect a significant difference between AN (n = 16) and HC groups (n = 16) on right-handed tasks with this measure [AN vs. HC (*t*_*right-parallel*_ = 3.75, *p* < .0001); AN vs. HC (*t*_*right-mirror*_ = 2.01, *p* = .04)].

### Statistical analysis

All data were analysed using SPSS Statistics 20 software. Independent *t*-tests were used to compare the two groups on demographic characteristics and eating disorder symptomatology variables (EDE-Q v.4 and BMI in kg/m^2^). Cohen’s *d* (mean1 - mean2/pooled standard deviation) was calculated to provide a measure of effect size where appropriate, with effect sizes of ≤ 0.2 defined as small, ≥ 0.5 defined as medium and ≥ 0.8 defined as large.

Independent *t-*tests compared the AN and HC groups on their scores on the self-report and experimental measures of body image perception, haptic perception and perfectionism, to explore differences on these measures.

Correlational analyses were performed separately on the clinical and HC data to explore possible relationships between variables within each group, using Pearson’s Product Moment correlation coefficients (*r*), or Spearman’s rho where data were not normally distributed, and focusing on relationships between the ALBA as a measure of body image distortion, with the haptic perception tasks and with the attitudinal measures.

Alpha was set at p < 0.05. Corrections to address the family-wise error rate in multiple analyses were carried out using Hochberg’s correction, as a less conservative method than Bonferroni’s correction method [47].

## Results

### Participant characteristics

Three HC participants were excluded because of possible caseness due to low BMI or clinical scores on the EDE-Q. One HC and four AN participants were excluded from the angle paradigm task analysis owing to left-handedness. The final data analysis included 28 in the HC group and 30 in the AN group. For analyses including the Angle Paradigm task, there were 27 in the HC group and 26 in the AN group.

### Demographic and clinical data

As expected, there was no significant difference between groups on age. Also as expected, the AN group had a significantly lower BMI than the HC group (*t*_*56*_ *=* 9.70, *p* <. 01), and scored significantly higher on the global scale of the EDE-Q (*t*_*56*_ *=* -10.53*, p* < .01).

### Self-report measures: body dissatisfaction & attitudinal factors

Descriptive statistics for the self-report measures are shown in Table 1.

**Table 1:** Means (M), standard deviations (SD), t-values, degrees of freedom (df) and effect sizes (d) of attitudinal measures of body image for Anorexia Nervosa (AN) patients and Healthy Controls (HC)

	**HC (n = 28)**		**AN (n = 30)**					
	***M***	***(SD)***	***M***	***(SD)***	***t***	***df***	***p***	***d***
**BMI**	23.36	(3.44)	16.42	(1.83)	9.51	40.5	<.001	2.59
**Body dissatisfaction variables**								
**Silhouettes**								
**With direction**	-1.26	(1.05)	-1.44	(2.91)	3.05	36.87	.38	0.08
**Without direction**	1.26	(1.05)	2.40	(2.17)	-2.57	42.56	.02	0.67
**Body image avoidance**								
**BIAQ Total score**	24.04	(6.77)	45.67	(15.66)	-6.91	40.05	<.001	1.8
**EDE-Q**								
**Shape concern**	1.33	(1.00)	4.36	(1.45)	-9.19	56	<.001	2.46
**Weight concern**	1.04	(0.91)	3.66	(1.57)	-7.84	47.18	<.001	2.06
**Global score**	0.80	(0.69)	3.84	(1.38)	10.76	43.27	<.001	2.81
**Perfectionism**								
**FMPS Total**	93.50	(13.37)	129.63	(21.33)	-7.78	49.19	<.001	2.05

(BMI = Body Mass Index; BIAQ = Body Image Avoidance Questionnaire; EDE-Q = Eating Disorder Examination Questionnaire; FMPS = Frost Multidimensional Perfectionism Scale.).

*Significance set at p =* .05.

#### Body dissatisfaction

Using the Silhouette task, all individuals in the HC group either were satisfied with their perceived size (25%; 7/28), or desired a smaller figure (75%; 21/28). 40% (12/30) of individuals in the AN group expressed a desire for a larger figure. A further 10% (3/30) of individuals in the AN group were satisfied with their perceived body size, thus 50% (15/30) expressed a desire for a smaller figure. As well as these between-group differences in the direction of desired change, the overall disparity between perceived and desired silhouette, ignoring the direction of desired body shape change, was significantly different, with the AN group significantly more dissatisfied with their bodies (*p* = .02).

On the BIAQ, the AN group showed significantly greater body dissatisfaction through behavioural expression (*t*_*40.05*_ = -6.91, *p* < .01). A similar finding was reported on the weight and shape subscales of the EDE-Q, with the AN group showing significantly greater Shape concern (*t*_*56*_ = -9.19, *p* < .01) and Weight concern (*t*_*47.18*_ = -7.84, *p* < .01).

#### Perfectionism

On the FMPS, the AN group scored significantly higher on the overall score, as expected (*t*_*49.19*_ *=* -7.78, *p* < .01). Analyses of subscales were not carried out, owing to lack of power. The perfectionism score was positively correlated with the exploration time on the Angle Paradigm Task, in the AN group only (*r* = .39, *p* = .03), meaning that the higher the perfectionism score, the longer the time taken to complete the task.

### Perceptual measures: haptic perception and body distortion

Descriptive statistics and pairwise comparisons for the experimental (perceptual) measures are shown in Table 2.

**Table 2:** Means (M), standard deviations (SD), t-values, degrees of freedom (df) and effect sizes (d) of perceptual measures of body image for Anorexia Nervosa (AN) patients and Healthy Controls (HC)

	**HC (n = 28)**		**AN (n = 30)**					
**Variable**	**M**	**SD**	**M**	**SD**	***t***	***df***	***p***	***d***
**Body size estimation (Light Beam Apparatus)**								
***With direction of inaccuracy***								
**% estimation accuracy - waist**	115.84	18.35	135.68	33.78	2.81	45.36	**.007**	0.74
**% estimation accuracy - hips**	120.45	16.06	137.50	44.19	1.98	36.10	.055	0.51
**% estimation accuracy - thigh**	104.36	19.10	136.61	53.66	3.09	36.68	**.004**	0.8
**% global estimation accuracy**	113.55	14.85	136.60	41.65	2.84	36.71	**.007**	0.74
***Without direction of accuracy***								
**% estimation accuracy - waist**	20.36	12.91	40.23	28.00	3.51	41.41	**.001**	0.92
**% estimation accuracy - hips**	21.56	14.48	43.22	38.40	2.88	37.58	**.007**	0.75
**% estimation accuracy - thigh**	15.65	11.43	44.60	46.99	3.27	32.66	**.003**	0.85
**% global estimation accuracy**	16.88	10.74	42.13	35.83	3.69	34.50	**.001**	0.96
**Excluding Left-handers**	**HC (n = 27)**		**AN (n = 26)**					
**Angle paradigm task**								
**All parallel tasks accuracy**	9.19	5.00	9.07	4.57	0.09	51.00	.463	0.03
**Exploration time**	11.69	4.40	24.60	13.41	4.67	30.15	**.0001**	1.33
**All mirror tasks accuracy**	6.60	2.53	6.25	2.76	0.47	51.00	.321	0.13
**Exploration time**	11.15	4.30	23.46	13.23	4.52	30.06	**.0001**	1.29
**All tasks accuracy**	7.89	3.15	7.66	3.09	0.27	51.00	.394	0.08
**Exploration time**	11.42	4.12	24.03	12.66	4.84	30.05	**<.0001**	1.38
**Right hand tasks**								
**Right parallel tasks accuracy**	9.34	5.30	10.01	4.53	0.49	51.00	.312	0.14
**Exploration time**	12.16	4.44	24.31	12.39	4.72	31.11	**<.0001**	1.34
**Right mirror tasks accuracy**	7.44	3.95	6.96	4.74	0.40	51.00	.347	0.11
**Exploration time**	11.15	4.27	23.86	12.52	4.91	30.54	**<.0001**	1.4
**Right hand tasks accuracy**	8.39	3.59	8.49	3.94	0.09	51.00	.463	0.03
**Exploration time**	11.65	3.97	24.08	11.60	5.18	30.58	**<.0001**	1.47
**Left hand tasks**								
**Left parallel tasks**	9.03	6.27	8.12	5.96	0.54	51.00	.295	0.15
**Exploration time**	11.22	4.66	24.88	14.93	4.46	29.67	**.0001**	1.27
**Left mirror tasks accuracy**	5.76	2.73	5.55	2.34	0.30	51.00	.384	0.08
**Exploration time**	11.16	4.76	23.05	14.83	3.90	29.91	**.0005**	1.11
**Left hand tasks accuracy**	7.40	3.59	6.84	3.19	0.60	51.00	.276	0.17
**Exploration time**	11.19	4.45	23.97	14.22	4.38	29.68	**.0001**	1.25

*Significance set at p =* .05.

#### BSE: body distortion

On the BSE task, using the Adjustable Light Beam Apparatus, two levels of analysis were undertaken, first looking at estimation accuracy without direction (pure accuracy), and secondly with direction (over or under-estimation). When the data were analysed without the direction of inaccuracy, the AN group overestimated their bodies more than the HC group overall, at waist, thighs and hips. The differences remained when the data were analysed with the direction of inaccuracy.

### Perceptual measures: haptic perception

Differences between groups on the Angle Paradigm task were explored using Independent samples *t* tests. No significant group differences were found on any conditions of the task. Group differences were found on total exploration time taken to complete the tasks, however, with the AN group taking significantly longer than the HC group (*t*_*30.05*_ = 4.84, *p* < .01).

Further analysis of the AN group was carried out to determine if severity of illness (as indicated by BMI and EDE-Q global score) was associated with performance on the Angle Paradigm task. No significant correlations were found between EDE-Q and haptic task performance. No significant correlations between BMI and Angle Paradigm tasks were found after Hochberg’s step up corrections for multiple testing. However, prior to correcting, a trend was found towards associations between the left-hand tasks and BMI, which might warrant further investigation in future studies.

### Exploring relationships with BSE

To investigate the relationship of perceptual versus attitudinal factors with BSE accuracy in AN, correlations between the variables in these two broad domains with BSE accuracy (with direction) were performed, using Pearson’s correlation coefficients for normally distributed variables and Spearman’s correlation coefficients for variables with skewed distributions. As multiple analyses were conducted, Hochberg’s step-up adjustment for significance was used within each domain (attitudinal or perceptual).

**Figure 1:**
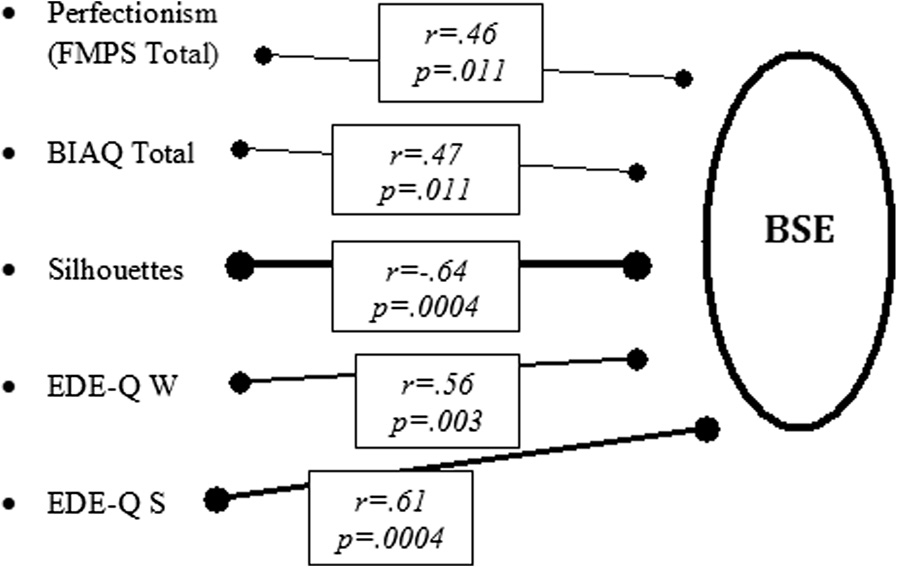
**Relationship between attidunal and perceptual factors with BSE in the AN group using Perason’s correlation coefficients (r).** Thickness of thw line indicates strength of the relationship. Significance level set at p= .05. (BSE= Body Size Estimation; AN= Anorecia Nervosa; FMPS= Frost Multidimensional Perfectionism Scale; BLAQ= Body Image Avidance Questionaire; EDE-Q W= Eating Disorders Examination, Weight Concern Subscale; EDE-Q S= Eating Disorders Examination Shape Concern Subscale).

A diagrammatic representation of the significant relationships of variables to body distortion (BSE, with direction of inaccuracy) in the AN group is displayed in Figure 1.

None of the other perceptual measures was significantly correlated with performance on the BSE task, whereas all the attitudinal measures significantly correlated with BSE.

A strong relationship was found between body dissatisfaction (with direction) and body distortion – the smaller the desired body shape, the greater the overestimation of body size. The score on the shape subscale of the EDE-Q was significantly related to the degree of overestimation – the higher the shape concern, the greater the overestimation. Scores on the BIAQ and FMPS were also significantly related to BSE – as the degree of body image avoidance increases, so does the degree of overestimation. Similarly, as the rating of perfectionist traits increases, so does the degree of overestimation.

Original and adjusted *p* values, using Hochberg’s step-up method are presented in Table 3.

**Table 3:** **Original and Hochberg step-up adjusted *p *** **values for correlations (Pearson’s correlations coefficient & Spearman’s Rank Correlation Coefficent: *r *** **&** ***r***_***s***_**) between attitudinal measures and BSE**

	**BSE with direction (over or under-estimating) *r & r***_***s***_** value**	**Original *p *** **value**	**Hochberg adjusted *p *** **value**
Silhouettes with direction (wanting to be smaller or larger)	.64	.0001	.0004
BIAQ (body image avoidance)	.47	.009	.011
FMPS (Perfectionism)	.46	.011	.011
EDE-Q Shape Concern subscale	.61	.0001	.0004
EDE-Q Weight Concern subscale	.56	.001	.003

(BSE = Body Size Estimation; BIAQ = Body Image Avoidance Questionnaire; FMPS = Frost Multidimensional Perfectionism Scale; EDE-Q = Eating Disorder Questionnaire).

Significance set at *p* = .05.

## Discussion

As expected, individuals with AN demonstrated higher levels of body image distortion as indicated by higher BSE errors on the Light Beam Apparatus, which confirms our hypothesis that AN individuals would overestimate their body size more than healthy controls. Additionally the AN group showed higher levels of body dissatisfaction than the HC group, through a number of self report measures, supporting previous findings [10].

Contrary to our hypothesis, previously reported haptic perception impairments in AN were not supported by our findings, with no significant difference between the AN and HC groups.

This difference may be accounted for by the increased time taken by the AN group to complete the task, which increased accuracy. This contrasts with Grunwald et al. [26] finding, where the groups took an equal amount of time. The AN group in this study were more inaccurate on the task than in Grunwald et al. [26] study, but the HC group were also more inaccurate, comparable with the AN group. This may point to a more impulsive and therefore inaccurate style within the HC group.

In accordance with our hypothesis the AN group reported significantly higher levels of perfectionism compared to controls.

In addition, associations between body image dissatisfaction, perfectionism, haptic perception and body image distortion were explored. There was no evidence to support a relationship between the haptic perception tasks and the body size estimation task, thus failing to support the hypothesis that fundamental haptic perception ability affects body distortion in AN. However, all the attitudinal, self-report measures were significantly correlated with body image distortion. Body dissatisfaction showed the strongest negative relationship – the smaller the desired silhouette than the actual, the greater the overestimation of body size, which supports Cash and Deagle’s [48] proposal that body overestimation may contribute to body dissatisfaction, though we can make no claims as to the direction of the relationship between the two. This links the concepts of dissatisfaction and distortion in individuals with AN, despite proposed separate neural correlates of each [49].

Overall, the positive relationships between body dissatisfaction and perfectionism with body size estimation in the AN group would suggest that attitudes (cognitions and affect) and behaviours are significantly related to body image distortion in AN.

The findings suggest that attitudinal factors and perfectionism are related to body distortion, but that there is no significant relationship between body distortion and haptic perceptual performance, at least as measured on a ‘neutral’ task involving haptic perception. This suggests that there are no fundamental haptic perceptual problems underlying body image disturbances in AN, but does not negate findings of sub-optimal visual perceptual performance in AN. Nor does it negate findings of parietal lobe dysfunction in AN (e.g. [29]), related to body image issues, which may involve difficulties in integrating sensory information from different modalities, rather than separate deficits in perception per se, as suggested by Case et al. [34] in their investigation of performance on the size-weight illusion task. In this context, it may be that haptic perception in individuals with AN is intact, at the fundamental level, but is overridden by an increased sensitivity to visual input, as seen in the rubber hand illusion [50], which then leads to a visual distortion of body image, and a lack of attention to proprioceptive or interoceptive information. Equally, it may be that body dissatisfaction impacts on the visual mental image of the body, which then also affects tactile perception when related to one’s own body, as suggested by Keizer et al. [10]. It will be of interest to use such “body-related” measures in future research to determine if a more salient focus does have an effect.

The strengths of this study are that, compared to previous work in the field, the two components of body image were measured separately, rather than treating them as a unitary concept. Confounding body dissatisfaction and body distortion has been argued to be a reason for mixed findings in the literature [49]. The haptic perception task provided a ‘neutral’ measure of fundamental somatosensory perception, divorced as far as possible from body attitudes. The BSE task, whilst not free from attitudinal biases, was intended to give as clear a picture of body image distortion as possible, including re-evaluation of the completed gestalt silhouette, and avoiding the use of distressing images of the individual. Coupled with self-report measures, this was intended to give as rounded a picture of body image disturbance as possible.

Some limitations must be considered. Firstly, the AN group spent significantly longer on the haptic perception task compared to the HC group, which may have allowed them to be more accurate than they would have been if there was a time limit imposed. Secondly, analyses of the angle paradigm task were found to be underpowered (post hoc analysis) which may explain non-significant findings.

Future research should replicate the Angle Paradigm Task performance in AN (and its subtypes) compared to HC with greater numbers as this study may have been underpowered in this domain. Given that the AN group took longer to complete this task, a time limit would be useful to determine any impact on accuracy. The use of both uni- and bi-manual tasks would assist in identifying performance related to sensory integration, and to haptic perception. A battery of haptic tasks with and without visual feedback would also clarify the picture of sensory processing in the disorder, as would comparison of ‘body-neutral’ tasks, with ‘own-body focused’ paradigms, likely to activate different bodily representations. Further exploration of the role of perfectionism in body image disturbance is warranted, and whether this relates to a subgroup of individuals with obsessive-compulsive traits. It will be useful to relate this to the specific perfectionism dimensions linked to eating disorder symptomatology, and then to body dissatisfaction and distortion in particular.

Clinically, this study highlights the role of cognitive behavioural interventions in modifying beliefs relating to body image and the use of exposure (behavioural tasks, mirror exposure) in addressing body size distortion (see [51-53]). Additionally, given the high levels of perfectionism and its association with body distortion, techniques which focus on acceptance (e.g. mindfulness; [54]) or on adapting CBT for clinical perfectionism [55] with specific reference to body image may be beneficial.

## Conclusion

Findings did not confirm the presence of fundamental haptic perceptual impairments in body image distortion in individuals with AN. Future work should explore whether or not previous findings in the literature demonstrating sub-optimal visual perceptual performance in AN could contribute to body image distortion. The findings did confirm the strong relationship between body image disturbance and cognitive-affective factors. This highlights the importance of continuing to focus on treatment interventions that target cognitive-affective biases and high levels of perfectionism as opposed to correcting underlying fundamental perceptual inefficiencies.

## Competing interests

The authors declare that they have no competing interest.

## Authors’ contributions

AW - Conceived and designed the protocol, conducted experiments, analysed the results, drafted the paper. RL - Conducted the experiments, took part in analysis, revision of the paper. VM – co supervised the project, participated in planning the study and revised the paper. KT – principal investigator, planned the study protocol, helped with the participant recruitment, supervised the project, edited drafts, submitted the paper. All authors read and approved the final manuscript.
